# Silence That Can Be Dangerous: A Vignette Study to Assess Healthcare Professionals’ Likelihood of Speaking up about Safety Concerns

**DOI:** 10.1371/journal.pone.0104720

**Published:** 2014-08-12

**Authors:** David L. B. Schwappach, Katrin Gehring

**Affiliations:** 1 Swiss Patient Safety Foundation. Zurich, Switzerland; 2 Institute of Social and Preventive Medicine (ISPM). University of Bern, Bern, Switzerland; The University of York, United Kingdom

## Abstract

**Purpose:**

To investigate the likelihood of speaking up about patient safety in oncology and to clarify the effect of clinical and situational context factors on the likelihood of voicing concerns.

**Patients and Methods:**

1013 nurses and doctors in oncology rated four clinical vignettes describing coworkers’ errors and rule violations in a self-administered factorial survey (65% response rate). Multiple regression analysis was used to model the likelihood of speaking up as outcome of vignette attributes, responder’s evaluations of the situation and personal characteristics.

**Results:**

Respondents reported a high likelihood of speaking up about patient safety but the variation between and within types of errors and rule violations was substantial. Staff without managerial function provided significantly higher levels of decision difficulty and discomfort to speak up. Based on the information presented in the vignettes, 74%−96% would speak up towards a supervisor failing to check a prescription, 45%−81% would point a coworker to a missed hand disinfection, 82%−94% would speak up towards nurses who violate a safety rule in medication preparation, and 59%−92% would question a doctor violating a safety rule in lumbar puncture. Several vignette attributes predicted the likelihood of speaking up. Perceived potential harm, anticipated discomfort, and decision difficulty were significant predictors of the likelihood of speaking up.

**Conclusions:**

Clinicians’ willingness to speak up about patient safety is considerably affected by contextual factors. Physicians and nurses without managerial function report substantial discomfort with speaking up. Oncology departments should provide staff with clear guidance and trainings on when and how to voice safety concerns.

## Introduction

Failures in communication among healthcare professionals (HCPs) remain a major root cause of adverse events [1]. Open and respectful communication about safety rule violations, potential mistakes and each other’s fallibilities is an essential resource to protect patients from harm, and to learn from errors as an individual, as a team, and as an organization. However, HCPs often report hesitating to speak up about their safety-related concerns [2], [3]. For example, in a recent study among HCPs in labor and delivery, only a minority of doctors, nurses and midwives reported sharing their full patient safety concerns with the errant colleague [4]. Organizational culture, personality traits and the interactions between them have been identified as important determinants of the propensity to speak up [5], [6]. Despite these stable factors, situation-specific conditions such as the clinical setting or the nature of the safety threat seem to influence the ad-hoc decision whether and how to voice concerns [7]. Willingness to speak up appears to fluctuate strongly in relation to context and social relationships between involved health care professionals. A better understanding of these influences on speaking up behaviors is required for the design of effective improvement activities such as training programs. There is, however, a paucity of research into the contextual factors that make speaking up about rule violations and errors in healthcare more or less likely [2].

Research from health care and other industries shows that differences in hierarchical status make speaking up difficult. Power discrepancies are an important inhibitor to speaking up in action teams, e.g., between nurses and surgeon in the operating room [8]. In a survey study among residents the decision to challenge a senior surgeon in the operating room was affected by the relationship and anticipated response of the superior [3]. Potential of patient harm has been identified as a major motivation for speaking up about safety concerns in labour and delivery whereas the fear to damage personal relationships and novelty of an alarming situation are strong barriers [9], [10]. Earlier experiences of speaking up which did not produce the desired outcome often results in decreased perceived effectiveness of speaking up and feelings of futility and resignation. Perceptions that voicing concerns will not make a difference are important barriers for future speaking up behaviour [6]. Finally, presence of patients or family in the situation has been reported to inhibit speaking up of health care workers towards their colleagues to avoid damage to the patient-provider relationship [2], [11]. In our previous qualitative research in oncology, nurses and doctors reported that they frequently experience situations which raise their concerns and require questioning, clarifying and correcting but that they occasionally decide to withhold concerns [11]. Oncology clinicians indicated that speaking up was related to the type of safety issue concerned. For example, medication safety concerns were easier to discuss whereas violations of hospital hygiene rules were rather difficult to voice. Clinicians typically felt strong obligation to prevent patient harm but this motivation competes with anticipated negative outcomes of speaking up (e.g., fears of punishment, damage of good relationships). Differences in hierarchical status or seniority between the involved persons seemed to influence self-reported speaking up behavior, but not necessarily in unidirectional and linear fashion.

This study investigates the self-reported likelihood to speak up about patient safety of clinicians in oncology and aims to clarify the effect of contextual factors on the likelihood of voicing concerns. We used brief clinical vignettes of errors and rule violations in cancer care to quantify the effect of situational context variables on professionals’ judgments of potential patient harm, perceived discomfort and their anticipated likelihood of speaking up. We examined whether health care workers’ personal characteristics, in particular profession and hierarchical status, affect their judgments of clinical situations requiring speaking up. Based on previous research, it was hypothesized that the likelihood of speaking up would fluctuate in relation to clinical safety issue [2], [11]. We expected that professionals of lower hierarchical status would be more likely to anticipate their withholding of voice but that this effect would be moderated by type of safety concerns. We hypothesized that clinicians would be hesitant to speak up in public forums, i.e., when other co-workers and patients or family are present, if power differentials are involved, and if the error/violation had been discussed before without effect. In contrast, we assumed that clinicians would be more willing to speak up about a coworker’s lapse as compared to negligent behavior and if the perceived potential for patient harm was high.

## Methods

### Ethics Statement

The study was exempted from full ethical review by the Ethics Committee of the Canton of Zurich (KEK-StV-Nr. 58/13).

### Survey instrument

The survey instrument was developed based on the literature and our prior in-depth qualitative research in the field [11]. In the part of the survey we report about herein respondents were presented four vignettes and asked to evaluate them. Vignettes are brief descriptions of fictive situations in which selected characteristics describing the objects to be judged by respondents are systematically manipulated [12]. The factorial survey approach is therefore well suited to study the contextual factors and conditions affecting judgment.

### Vignettes

**Figure 1:**
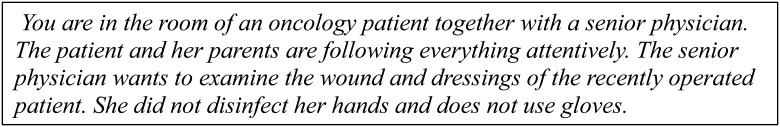
Example vignette.

The vignettes described hypothetical clinical situations in which a staff member (actor) makes an error in patient care. Immediate action by bystanders is required to avoid potential patient harm. The vignettes consisted of a clinical frame (“storyboard”) in which binary variables (attributes) were embedded and systematically manipulated. The clinical frames were derived from reports obtained in qualitative interviews with oncology staff [11] and involved: an error in checking a prescription (frame A), a missed hand disinfection (frame B), a safety rule violation in medication preparation, (frame C), and a safety rule violation in lumbar puncture (frame D) (see Appendix S1). Seven attributes with two binary levels each were used to operationalize and test our hypothesis about factors affecting HCPs’ decisions to speak up: profession of actor (nurse/doctor) and seniority of actor (high/low) were chosen as to illustrate power differentials; number of staff present in the situation, i.e., privacy (few/many) and patient or relative present and attentive (yes/no) were used to describe public vs private forums; the latter variable was also chosen to indicate a potentially irritating situation for patients and thus a strong motivation for withholding voice among health care workers; repeated occurrence of the same violation (yes/no) aimed to signal potential ineffectiveness of speaking up; negligent behavior of the actor (yes/no) was chosen to indicate a potentially difficult social situation and response; level of potential patient harm (high/low) was used to indicate level of threat. Each of the four clinical frames contained a different subset of three out of these seven binary attributes. For example, different versions of the missed hand hygiene scenario were prepared which described either a nurse or a doctor being non-compliant, the situation taking place “in public” with many as compared to a private situation with no further staff present, and the patient being distractive or vigilant to the rule violation (Figure 1 presents an example). Vignettes differ in the levels of attributes and differences in attribute levels are used to explain variance in respondents’ judgments. In a full factorial design, the number of attributes and levels results in 8 possible combinations (levels^attributes^ = 2^3^) and thus versions of each vignette. With the four different clinical frames, there were 32 different vignettes in total (8 versions of each of the 4 clinical frames). Eight survey versions were prepared and contained one randomly selected vignette of each clinical frame (A–D). In summary, each participant provided judgments of one version of each type of error/rule violation.

### Outcome measures

Each vignette was followed by the same survey questions measured on a 7-point Likert-like scale: (1) how great the potential for patient harm is (1 = very low, 7 = very high); (2) how uncomfortable they would feel to speak up (1 = very comfortable, 7 = very uncomfortable); (3) how likely it would be that they speak up, using words or gestures (1 = very unlikely, 7 = very likely); (4) whether it would be difficult to decide how to react (1 = very easy, 7 = very difficult). Respondents were also asked to judge whether the situation is realistic on their job (1 = not realistic at all, 7 = very realistic).

Vignette stories were approved by 8 clinical oncology experts (specialist nurses and oncologists from adult and pediatric oncology). Experts were asked to check and approve: whether the cases could potentially occur as described and whether they are realistic and clear; whether any important clinical information is missing which would help respondents to interpret the situation (e.g., in the lumbar puncture scenario, clinicians recommended including the information that the patient is currently receiving chemotherapy to signal increased risk of bleeding); whether the scenario would be appropriate for both, adult and pediatric oncology respondents; and whether they feel that staff could respond to the survey items. The survey was pre-tested with clinical staff from non-participating hospitals (n = 30). After completing the survey, they were asked to report on comprehensibility, realism of the scenarios, the answerability of the questions, and to mark any ambiguous wording. Few changes were made to survey wording and layout.

### Personal characteristics

Responders were asked to provide personal information. This included age, gender, years of professional experience, years of work experience in oncology, whether they work on ward or in ambulatory infusion units. Doctors and nurses were asked to provide information about their job status. This information was used to categorize responders as staff with (attending/senior/chief/head nurse) and without (nurse in training/nurse/resident) managerial functions. Managerial function is a measure of higher hierarchical status and less proximity and time spent in direct patient care.

### Sample

Eight Swiss hospitals participated with nine oncology departments. These included two university hospitals with adult oncology units, two paediatric university hospital departments, and five regional hospitals. One hospital participated with the adult and the paediatric oncology units. All doctors and nurses working at the oncology wards or ambulatory were included (sample I: n = 759). The mean number of HCPs per hospital was 84 (range: 13–304). Department heads or study mentors informed about the study at morning rounds, staff meetings or training sessions. They distributed the survey and asked staff to participate. HCPs received the survey together with a pre-paid envelope and a chocolate bar. Survey versions were randomized and distributed to hospitals. Due to anonymity, no individual reminders could be sent but HCPs were reminded to participate at the group level. In addition to the paper-based form, the survey was also distributed as an online-survey to oncology nurses registered with the Swiss Oncology Nursing Association (sample II: n = 796). This survey started after the field phase of the paper-based survey was completed. Members were asked to participate via email and sent an individualized and secured online form for participation. To minimize sample overlap, addresses consisting of sample I hospital domains were deleted from the register prior to invitation. Further, individuals in sample II indicated whether they had participated in the paper survey at survey start and were then excluded from online-participation. Paper and online survey were designed as identical as possible (e.g., page breaks). Non-responders to the online survey received one reminder. Return of the survey was considered informed consent.

### Data Analysis

Descriptive statistics are used to report survey responses. We examined the variance and correlations among respondents’ likelihood of speaking up ratings of the four vignettes. T-tests were conducted to examine differences in mean ratings between groups of staff (nurses vs doctors; staff with vs. without managerial function). Multiple regression analysis was used to model the likelihood of speaking up as outcome of vignette attributes, responder’s evaluations of the situation and personal characteristics. The unit of analysis is the judgment provided to each survey question in response to a vignette, and not the individual respondent. We used robust (sandwich) estimators of variance to relax the assumption of independence of observations. All tests were two-sided and a p-value≤0.05 was considered significant.

### Sample size and power considerations

In analysis of factorial survey data, the unit of analysis is the vignette. Statistical power is determined by the number of respondents and the number of vignettes judged by each respondent (sample size = number of respondents×vignettes per respondent) [12].

We estimated that for the planned multiple regression analysis with up to 35 predictor variables (vignette attributes, vignette ratings, respondents’ characteristics and potential interactions), a small effect size of 0.02, and power of 0.95 a sample size of 1906 judgments would be required [13]. This number translates to 477 respondents as individuals rate four vignettes.

## Results

In total, 1013 HCPs returned the completed survey for a response rate of 65% (paper survey: 69%; online survey: 61%). The eight survey versions were evenly distributed across participants in both the paper and the online survey. Table 1 provides participants’ characteristics. The situations described in the vignettes were on average perceived as being realistic by respondents (mean realism score across all vignettes 4.2 (SD 2.03)). We observed several indications that participants thoroughly evaluated the vignettes and adjusted their ratings sensitively in the expected direction. For example, the potential of harm rating was considerably affected by the drug involved in the vignettes describing a failure to check a prescription (4.8 for premedication vs. 6.4 for vincristine, p<0.001). The six pairwise correlations among the likelihood of speaking up ratings for the four vignettes were significant but small (r_vignettes 1and2_ = 0.31; r_vignettes 1and3_ = 0.29; r_vignettes 1and4_ = 0.29; r_vignettes 2and3_ = 0.25; r_vignettes 2and4_ = 0.27; r_vignettes 3and4_ = 0.26).

**Table 1:** Characteristics of survey responders (n = 1013).

Characteristic	Responders	
	n	%
Sample		
Sample 1 (paper survey)	525	52
Sample 2 (online survey)	488	48
Female gender	800	80
Age, mean (SD) years	40 (11)	
18–25 years	94	9
26–40 years	441	44
41–55 years	394	39
56–65 years	73	7
Profession		
Doctor	131	13
Resident	61	6
Senior	38	4
Head senior	23	2
Chief	9	1
Nurse	780	79
Nurse in training	22	2
Nurse	570	58
Head nurse (with managerial function)	151	15
Nursing expert	37	4
Other (e.g. pharmacist)	71	7
Years of practice in oncology, mean (SD) years	9 (7)	
1–5 years	359	38
6–10 years	266	28
11–25 years	290	31
>25 years	26	3

### Differences between clinical frames and vignettes

Across all vignettes and clinical frames, respondents reported a high likelihood of speaking up but the variation between and within types of errors/rule violations (frames) was substantial: Across the vignettes of each frame, between 68% (missed hand disinfection) and 90% of participants (rule violation medication preparation) said they would speak up in the scenario presented. Based on the information presented in the vignettes, the fraction of responders who reported they would speak up varied between 74%−96% (error in checking a prescription), 45%−81% (missed hand disinfection), 82%−94% (rule violation in medication preparation), and 59%−92% (rule violation in lumbar puncture). In other words, the vignette characteristics accounted for – on average – a 25% change in the fraction of responders who said it would be likely or very likely for them to speak up. Table 2 reports mean ratings of harm, discomfort, decision difficulty and likelihood of speaking up for the four frames. Analysis of variance revealed that for all outcomes measures mean ratings differed significantly between clinical frames. The two vignettes which were assigned the lowest mean likelihood of speaking up were both describing a senior doctor forgetting hand disinfection (mean scores of 3.8 and 4.8 respectively).

**Table 2:** Mean ratings of harm, discomfort, decision difficulty and likelihood of speaking up across vignettes, by clinical frame.

	Mean rating (SD)#				
Clinical Frame^+^	Harm	Discomfort	Decision difficulty	Likelihood	% likely tospeak up*
A: Error in checking a prescription	5.67	3.16	2.43	6.15	89%
	(1.51)	(1.99)	(1.64)	(1.35)	
B: Missed hand disinfection	5.68	3.85	3.27	5.14	68%
	(1.31)	(2.12)	(1.94)	(1.90)	
C: Rule violation medication preparation	5.79	2.39	2.10	6.18	90%
	(1.37)	(1.79)	(1.54)	(1.34)	
D: Rule violation lumbar puncture	5.60	3.18	2.75	5.69	79%
	(1.45)	(2.24)	(1.92)	(1.77)	
Total	5.68	3.14	2.64	5.79	81%
	(1.41)	(2.10)	(1.92)	(1.66)	
p (differences between clinical frames)	0.0216	<0.001	<0.001	<0.001	<0.001

N = 1013 participants; n = 4052 vignette evaluations;

# higher values indicate higher levels of potential for patient harm, feeling less comfortable to speak up, higher decision difficulty, and higher likelihood of speaking up.

*responders with rating >4; ^+^mean across the 8 vignettes within each frame.

### Differences between groups of staff

**Figure 2:**
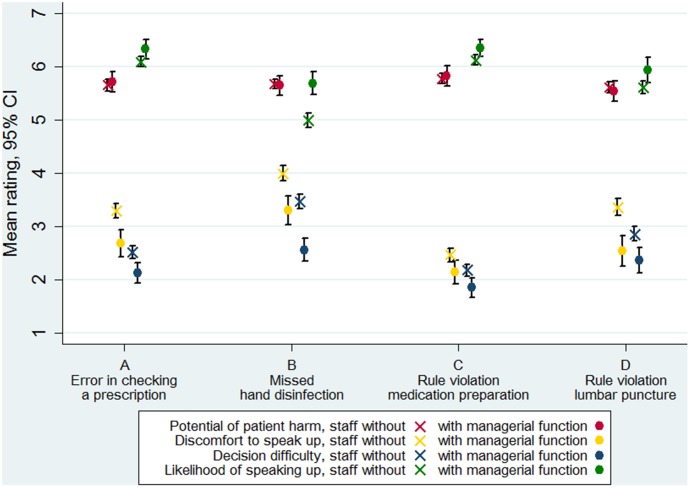
Mean ratings of potential of patient harm, discomfort to speak up, decision difficulty and likelihood of speaking up for the four clinical frames by managerial function of respondent.

Mean potential for patient harm ratings were slightly higher for nurses as compared to doctors (5.77 vs. 5.39, p<0.001) but did not differ according to seniority or place of work. We also found systematic differences between staff with and without managerial functions. Staff without managerial function systematically provided significantly higher levels of decision difficulty and discomfort to speak up across all vignettes though the magnitude in difference was affected by type of error or rule violation (clinical frame) (Figure 2). Contrary, the potential of harm ratings were in well concordance between staff with and without managerial function. Reported likelihood of speaking up was significantly higher among staff with managerial function in the missed hand disinfection vignettes. Staff with managerial function was nearly half as likely as compared to staff without managerial function to report discomfort about speaking up across all vignettes (Table 3, Odds ratio = 0.55, p<0.001). The odds ratio of reporting a high likelihood of speaking up across all vignettes was 1.71 (p<0.001) for staff with as compared to staff without managerial function.

**Table 3:** Association of respondents’ managerial function and dichotomized ratings of harm, discomfort, decision difficulty and likelihood of speaking up across vignettes, by clinical frame.

	Odds ratios* of staff with vs without managerial function							
	Harm		Discomfort		Decision difficulty		Likelihood of speaking up	
Clinical Frame	Odds Ratio	p	Odds Ratio	p	Odds Ratio	p	Odds Ratio	p
A: Error in checking a prescription	1.23	0.300	0.59	0.002	0.39	0.001	1.55	0.106
B: Missed hand disinfection	0.95	0.817	0.53	<0.001	0.41	<0.001	2.54	<0.001
C: Rule violation medication preparation	0.90	0.615	0.62	0.030	0.63	0.105	1.32	0.298
D: Rule violation lumbar puncture	0.92	0.629	0.43	<0.001	0.69	0.063	1.38	0.106
Total	0.99	0.959	0.55	<0.001	0.52	<0.001	1.71	<0.001

N = 1013 participants; n = 4052 vignette evaluations;

*Measures (harm, discomfort, decision difficulty, reported likelihood of speaking up) are dichotomized with cutoff >4.

### Predictors of the reported likelihood of speaking up

Table 4 reports the results of the multiple regression analysis. This analysis reveals that the reported likelihood of speaking up was strongly affected by contextual factors embedded in the vignettes: As hypothesized, respondents were less likely to speak up towards the senior physician who signs an errant medication order in a public forum when several other HCPs are present and more likely when the order involves a high-risk drug (vincristine). In line with our assumptions, participants’ reported likelihood of speaking up regarding a missed hand disinfection was negatively affected by patients’ attentiveness to the situation and when the actor was a physician. The likelihood of speaking up towards nurses who violate the double check rule in medication preparation was only affected by the repeat occurrence of this rule violation but not by seniority of the involved nurses, contrary to our hypothesis. However, speaking up against the rule violation in lumbar puncture was strongly determined by seniority of the actor with respondents being less likely to speak up towards a senior physician as expected. Across clinical frames, the level of perceived potential harm, anticipated discomfort, and decision difficulty considerably impacted on the likelihood of speaking up. Male gender, younger age, being a nurse, and working on ward were also significantly associated with a lower expressed likelihood of speaking up.

**Table 4:** Results of multiple regression analysis with reported likelihood of speaking up as outcome.

	Coefficient	95% CI	p
Clinical frame, basel level: A (Error in checking a prescription)			
B: Missed hand disinfection	−0.273	−0.908, 0.361	0.399
C: Rule violation medication preparation	−0.274	−0.787, 0.239	0.295
D: Rule violation lumbar puncture	−0.061	−0.668, 0.545	0.843
Attributes of clinical frame A (Error in checking a prescription)			
Several staff present	−0.370	−0.515, −0.226	<0.001
Negligent behavior of the actor	−0.061	−0.206, 0.084	0.412
Potential harm	0.193	0.038, 0.347	0.014
Attributes of clinical frame B (Missed hand disinfection)			
Several staff present	0.026	−0.164, 0.215	0.790
Patient present and attentive	−0.276	−0.465, −0.088	0.004
Profession of actor (physician vs. nurse)	−0.206	−0.396, −0.016	0.034
Attributes of clinical frame C (Rule violation medication preparation)			
Negligent behavior of the actor	0.064	−0.090, 0.218	0.417
Seniority of the actor	0.038	−0.115,0.191	0.628
Repeated occurrence	−0.284	−0.438, −0.131	<0.001
Attributes of clinical frame D (Rule violation lumbar puncture)			
Seniority of the actor	−0.332	−0.527, −0.138	0.001
Patient present and attentive	−0.143	−0.332, 0.047	0.141
Repeated occurrence	0.063	−0.131, 0.256	0.525
Level of harm rating	0.178	0.143, 0.213	<0.001
Level of discomfort rating	−0.149	−0.181, −0.116	<0.001
Decision difficulty rating	−0.296	−0.336, −0.255	<0.001
Male gender	−0.407	−0.532, −0.282	<0.001
Age, years	0.014	0.008,0.020	<0.001
Nurse	−0.171	−0.281, −0.060	0.002
Working on ward	−0.184	−0.273, −0.095	<0.001
Managerial function	0.024	−0.080,0.128	0.654
Years of practice in oncology	−0.006	−0.014, 0.003	0.188
Constant	6.502	6.032, 6.972	<0.001
R^2^	0.367		
Cohen’s F^2^	0.580		
overall model p			<0.001
n vignette evaluations	3636		
N participants	909		

## Discussion

This study investigates the likelihood of speaking up about patient safety in oncology. Physicians and nurses in our study perceived a high level of potential patient harm associated with the four errors and rule violations (clinical frames). On average, participants reported the lowest likelihood of speaking up about a missed hand disinfection. Our results support our hypothesis that speaking up behaviors are considerably affected by situational factors. We found large variability in the reported likelihood of speaking up across and within types of errors. The fraction of responders who said they would speak up ranged between 45%–96%, depending on type of incident and vignette specifications. Moreover, all measures of potential patient harm, discomfort, and decision difficulty differed significantly between the types of rule violations/errors.

Our results provide evidence that HCPs of lower hierarchical status find it much more difficult to decide and perceive considerably higher levels of discomfort associated with speaking up. The regression analysis reveals that staff without managerial function is not *per se* hesitant to speak up, but that it is the difficult emotions connected to the behavior that makes speaking up less likely. As reported by others, potential harm was a strong predictor for likelihood of speaking up in our study [10]. However, the magnitude of its effect only slightly exceeded that of the discomfort rating in regression analysis. These results show impressively the difficult trade-offs clinicians face when deciding to speak up and have important consequences: First, clinical leaders need to be made aware of the emotional demands of speaking up prevalent among their subordinates and that their own perception of the challenges associated with speaking up is likely to differ from those of lower hierarchical status. A similar finding has been reported for safety climate perceptions which tend to be more positive among senior managers [14]. Second, decision difficulty and anticipated distress may be modifiable by team training and guidance about which clinical situations warrant speaking up. Our results suggest such trainings to be as close to reality as possible to take into account the relevance of situational conditions conducive of “silence”. Our vignettes may serve as valuable triggers in team discussions or could be used for role-play in team trainings.

A number of attributes used to characterize the vignettes significantly affected oncology clinicians’ anticipated likelihood of speaking up. Regression analysis indicates that the presence of “others” plays an important role for the decision to voice concerns in some contexts but not in others. Clinicians avoided *public forums* to raise their concerns and were more likely to “voice behind closed doors”, a finding that has been reported from outside the healthcare setting [7]. One motivation is to avoid compromising the actor in public, risking to be humiliated in front of peers in response, and to mitigate the risks of challenging a supervisor or coworker. Preserving trust of patients in clinicians is a strong motivation to withhold voice in the presence of patients. In our study, responders were very reluctant to point coworkers to a missed hand disinfection when the vignette suggested that patients would follow this communication. A considerable fraction indeed indicated they would withhold voice, even at the price of potential patient harm. Errors in clinical procedures, hygiene violations, and communication errors with “a now-or-never timeframe” do, however, occur frequently in the presence of patients or relatives. HCPs are then faced with the difficult maneuver to correct fallibilities and prevent harm without undermining the patient relationship. We suggest that leaders provide guidance, in particular for younger and less experienced staff when and how to speak up under such conditions. The use of gestures and “stop-words” may be useful to intervene safely but more research is clearly needed to explore effective approaches.

The repeat occurrence of a rule violation affected speaking up likelihood in the medication double check frame, but not in the lumbar puncture vignettes. With all else being equal, respondents were more likely to withhold voice when they had been instructed that the violation of the double check had been observed and discussed before. Obviously, respondents “learned the lesson” that speaking up would be ineffective and not worth the efforts fast. The adaption to rule violating behavior and the “normalization of deviance” have been identified as genuine risks to patient safety [15], [16]. Amalberti describes how deviances from safety rules occur, stabilize, and become routine if they are not actively managed by healthcare organizations [17]. Our study suggests that HCPs forecast their speaking up behaviors’ adaption to resistant rule violations and that these processes may spread to a “culture of silence” in the long-term.

Our study has some weaknesses. The main limitation is that we did not observe speaking up but asked subjects to report their anticipated behaviors. Thus, our speaking up estimates are likely to be subject to hypotheticality and social desirability bias. Previous research into clinical decision making shows, that judgments made in response to vignettes are often similar to those made with actual patients [18]–[21]. To the contrary, actual speaking up decisions are likely to be affected by factors we could not simulate in our vignette approach. For example, time pressure and social relationships have been reported to be important barriers to speaking up [9], [11]. Participants in our study could make deliberate decisions after considering the potential risks and benefits of speaking up, something that is often not possible in clinical care. Research into affective forecasting shows that subjects often fail to predict their emotional response to future events and typically overestimate the intensity and duration of their emotional response due to ‘impact bias’ [22]. In effect, we cannot rule out that responders in our study over- or underestimated their own willingness to voice and we do not know whether participants’ hypothetical behaviors correlate with their actual behaviors. A second limitation is that nurses are overrepresented in the sample due to the sampling strategy. While systematic differences in outcome variables between nurses and physicians exist, there were no differences between nurses approached via hospitals (sample I) and those included in the professional membership file (sample II). As we have no data about non-responders or about the distribution of characteristics in the entire oncology staff population we cannot estimate how representative our sample is. The strengths of this study are the relatively large sample size and the high response rate to the survey. In addition, we approached staff from a heterogeneous group of hospitals. The low correlations between the vignette ratings of each individual also provide some evidence that situation-specific context is important and that responders adjusted their ratings in response to the information provided. Finally, the use of a full-factorial experimental design allowed us to estimate all possible combinations of contextual factors without contamination by other factors.

In conclusion, clinicians’ willingness to speak up about errors and rule violations was generally high but differed strongly according to type of error and rule violation. Physicians and nurses in oncology, in particular those without managerial function, reported substantial discomfort with speaking up. The results offer important insights into the factors affecting the likelihood of speaking up and, after being confirmed in further research, could be used to design trainings for oncology staff.

## Supporting Information

Appendix S1 **Attributes, attribute levels and vignette phrasing (italics).**
(DOCX)
