# Supplementary material for: Silence That Can Be Dangerous: A Vignette Study to Assess Healthcare Professionals’ Likelihood of Speaking up about Safety Concerns
Source: PLoS One. 2014 Aug 12;9(8):e104720. doi: 10.1371/journal.pone.0104720 (PMC4130576; doi:10.1371/journal.pone.0104720)
Supplement: Appendix S1 — Attributes, attribute levels and vignette phrasing (italics). (DOCX) [file pone.0104720.s001.docx]

**Appendix 1:** Attributes, attribute levels and vignette phrasing (italics)

| **Frame A: Error in checking a prescription** | | |
| --- | --- | --- |
| Number of staff present | | |
|  | Few | *You are in a meeting with the senior physician in his office.* |
|  | Many | *You are in a meeting with several nurses and physicians on ward.* |
| *By chance you notice that the prescription of* [level of potential harm] *is unusually high in an order form on the table.* | | |
| Level of potential harm | | |
|  | Low | *Premedication* |
|  | High | *Vincristine* |
| Negligent behavior of the actor | | |
|  | No | *The prescription has already been signed by the senior.* |
|  | Yes | *The senior signs some urgent orders of a resident. The senior only glances over the orders. He gives his signature “blind” on the wrong place in the paper form. He says “…it is certainly ok. I have to carry on here.”* |

| **Frame B: Missed hand disinfection** | | |
| --- | --- | --- |
| Number of staff present | | |
|  | Many | *You are on ward round together with nurses and doctors in the room of an oncology patient.* |
|  | Few | *You are together with* [Profession of actor] *in the room of an oncology patient.* |
| Patient present and attentive | | |
|  | No | *The patient is talking to her parents and is inattentive at the moment.* |
|  | Yes | *The patient and her parents are following everything attentively.* |
| *The* [actor] *wants to examine the wound and dressings of the recently operated patient.* | | |
| Profession of actor | | |
|  | Nurse | *Head nurse* |
|  | Doctor | *Senior physician* |
| *She did not disinfect her hands and does not use gloves.* | | |

| **Frame C: Rule violation in medication preparation** | | |
| --- | --- | --- |
| *You are in a room where medications are just being prepared. As time is short, nurses are stressed out. You notice that the double check obligatory for some high-risk drugs is being omitted by* [actor]*.* | | |
| Seniority of the actor | | |
|  | Low | *Nurses* |
|  | High | *Head nurse* |
| Repeated occurrence of the same violation | | |
|  | No | *--* |
|  | Yes | *This had happened a few weeks before and your colleague pointed the* [Actor] *to the importance of the check.* |
| Careless behavior of the actor | | |
|  | No | *--* |
|  | Yes | [Actor] *says to her colleague: „Come on, that will be ok, I am in a hurry.“* |

| **Frame D: Rule violation in lumbar puncture** | | |
| --- | --- | --- |
| *You prepare the lumbar puncture of a chemotherapy patient with [Actor].* | | |
| Seniority of the actor | | |
|  | Low | *A resident* |
|  | High | *A senior physician* |
| Patient present and attentive | | |
|  | No | *The patient is still at his room.* |
|  | Yes | *The patient is already prepared.* |
| *You recognize that there are no fresh* coagulation parameters and *platelet count available for the patient. Nevertheless, the* [Actor] *wants to proceed with the puncture because everybody is under time pressure and the daily schedule would get messed up otherwise.* | | |
| Repeated occurrence of the same violation | | |
|  | No | *--* |
|  | Yes | *You heard that a similar situation occurred recently. The puncture had been performed at this occasion, despite the objections of a staff member.* |
